# Sex‐Dependent Influence of Major Histocompatibility Complex Diversity on Fitness in a Social Mammal

**DOI:** 10.1111/mec.70058

**Published:** 2025-07-26

**Authors:** Nadine Schubert, Hazel J. Nichols, Francis Mwanguhya, Robert Businge, Solomon Kyambulima, Kenneth Mwesige, Joseph I. Hoffman, Michael A. Cant, Jamie C. Winternitz

**Affiliations:** ^1^ Department of Animal Behavior Bielefeld University Bielefeld Germany; ^2^ Department of Biosciences Swansea University, Singleton Campus Abertawe UK; ^3^ Banded Mongoose Research Project Queen Elizabeth National Park, Kasese District Rubirizi Uganda; ^4^ Department of Evolutionary Population Genetics, Faculty of Biology Bielefeld University Bielefeld Germany; ^5^ Faculty of Biology, Center for Biotechnology (CeBiTec) Bielefeld University Bielefeld Germany; ^6^ British Antarctic Survey Cambridge UK; ^7^ Joint Institute for Individualisation in a Changing Environment (JICE) Bielefeld University and University of Münster Bielefeld Germany; ^8^ Centre for Ecology and Conservation, University of Exeter, Penryn Campus Penryn UK; ^9^ Evolutionary Immunogenomics Institute for Animal Cell and Systems Biology, University of Hamburg Hamburg Germany

**Keywords:** antagonistic selection, banded mongoose, lifetime reproductive success, MHC, *Mungos mungo*

## Abstract

Parasite infections affect males and females differently across a wide range of species, often due to differences in immune responses. Generally, females tend to have stronger immune defences and lower parasite loads than males. The major histocompatibility complex (MHC) plays a crucial role in the adaptive immune response, and extensive research has explored how variation in this region influences infection and fitness outcomes. However, studies of sex‐specific relationships between MHC variation and infection are scarce, perhaps because MHC genes are located on the autosomes, which are shared by both sexes. Here, we provide evidence of sexually antagonistic selection in a wild, group‐living mammal—the banded mongoose. Using genetic and life history data collected from over 300 individuals across 25 years, we found that both MHC class I (MHC‐I) and MHC class II (MHC‐II) diversity influence lifetime reproductive success differently in males and females. Specifically, higher MHC diversity is linked to increased fitness in males but decreased fitness in females. Furthermore, MHC diversity did not differ between the sexes, indicating an unresolved genetic sexual conflict. Our findings demonstrate that sexually antagonistic selection acts on the MHC and may operate across both MHC classes but differently. This study contributes to the growing body of evidence that sex is a significant factor in shaping host immunity and fitness.

## 1 Introduction

Parasites can strongly reduce individual fitness, influencing both longevity and reproductive success (e.g., Leivesley et al. 2019; Pedersen and Greives 2008; Tompkins and Begon 1999). The immune system, which detects and eliminates both macroparasites (e.g., helminths, arthropods, protozoa) and microparasites (e.g., viruses, bacteria, fungi), plays a central role in determining parasite burden. Variation in immune effectiveness shapes an individual's susceptibility to infection and, by extension, their fitness (e.g., Marzal et al. 2005). Some individuals consistently carry higher parasite loads due to factors such as age and genetic heterozygosity (Budischak et al. 2023; Mitchell et al. 2017; Hoffman et al. 2014), but one of the most striking predictors of immune variation is biological sex.

Immune responses often differ between males and females. Across vertebrates, females typically show lower parasite loads than males (Moore and Wilson 2002; Zuk and McKean 1996), a pattern ultimately attributed to different life history strategies that select for increased investment in immunity in females to increase their number and quality of offspring and increased investment in secondary sex traits and competitive ability in males to increase their mating opportunities (Rolff 2002; Zuk 1990; Zuk and Stoehr 2002). In vertebrates, immune dimorphism is commonly attributed to the immunosuppressive effects of testosterone in males and the immunoenhancing effects of oestrogen and progesterone in females (reviewed in Roved et al. 2017). These hormonal influences can generate sex‐specific selection pressures on immune genes, particularly those involved in pathogen recognition. As a result, sexually antagonistic selection could favour different optimal levels of immune gene diversity in males and females, with higher diversity potentially benefiting males more than females (Roved et al. 2017).

A crucial component of the adaptive immune response against parasites is the major histocompatibility complex (MHC). The MHC is a gene complex that is found in virtually all jawed vertebrates (Kaufman 2018) and is consistently one of the most polymorphic regions of their genomes, as shown in humans, chickens, pigs, and mice (1000 Genomes Project Consortium 2010; International Chicken Polymorphism Map Consortium 2004; Nicod et al. 2016; Tong et al. 2023). MHC molecules encode cell‐surface glycoproteins that are crucial for the initiation of the adaptive immune response (Bjorkman et al. 1987). Classical MHC molecules bind and present self and foreign peptides to professional immune cells which can cause further activation of the immune response (Knapp 2005). The classical MHC loci are subdivided into MHC class I (MHC‐I) and MHC class II (MHC‐II). MHC‐I molecules are present on nearly all nucleated cells and mainly present peptides originating from intracellular sources (e.g., self‐derived peptides and peptides originating from viruses or other pathogens that have entered the cell), while MHC‐II molecules are present on professional antigen‐presenting cells (APCs, including macrophages, B cells and dendritic cells), and present peptides originating mostly from exogenous sources (e.g., derived from bacteria or parasite particles that have been engulfed by the cell) (Neefjes et al. 2011). Because each MHC allele binds only a subset of peptides (Rammensee et al. 1999), an individual's allelic diversity at the MHC determines the breadth of pathogen detection.

Several hypotheses attempt to explain how MHC diversity influences fitness. *The maximal diversity hypothesis* proposes that individuals with more MHC alleles can recognise and eliminate a wider range of pathogens, thereby enhancing immune defence and reducing parasite loads and diversity (Doherty and Zinkernagel 1975). Empirical support for this hypothesis comes from studies showing a negative relationship between MHC diversity and parasite load, and a positive link between MHC diversity and fitness (Agudo et al. 2012; Biedrzycka et al. 2018; Bonneaud et al. 2004; Kloch et al. 2010; Radwan et al. 2012).

By contrast, the *optimal diversity hypothesis* suggests that too many MHC alleles can be detrimental, as the increased variety of peptides presented may lead to T‐cell depletion or self‐reactivity, reducing immune efficiency (Wegner et al. 2003; Nowak et al. 1992; Woelfing et al. 2009; but see Borghans et al. 2003). This hypothesis predicts an intermediate number of MHC alleles as optimal. However, direct immunological evidence for this model is mixed (Brown et al. 2024; Krishna et al. 2020; Migalska et al. 2019) and a number of studies investigating the link between MHC diversity and parasite load on fitness have failed to support either the maximal or the optimal diversity hypothesis (Harf and Sommer 2005; Meyer‐Lucht and Sommer 2005; Radwan et al. 2012; Sepil, Lachish, Hinks, and Sheldon 2013; Sepil, Lachish, and Sheldon 2013; Westerdahl et al. 2012). Consequently, the evolutionary mechanisms linking MHC diversity, parasite load, and fitness remain unresolved.

One reason for these inconsistent findings may be analyses that assume that MHC effects are the same in both sexes. However, evolutionary outcomes of MHC variation can differ by sex, even for autosomal genes. A handful of studies have begun to explore this, revealing sex‐specific associations between MHC diversity and survival or reproductive success. For instance, in alpine chamois, only males showed increased longevity with MHC‐II DRB heterozygosity, controlling for background heterozygosity (Schaschl et al. 2012). In great reed warblers, MHC‐I diversity was positively associated with male reproductive success but negatively associated with that of females—suggesting unresolved sexual conflict over immune gene diversity (Roved et al. 2018). Yet most existing work focuses on a single MHC class, thereby failing to capture the full scope of functionally relevant immunogenetic variation.

To better understand how natural selection shapes immune gene variation differently in males and females, more comprehensive studies are needed across both MHC classes. A long‐term study of banded mongooses ( *Mungos mungo* ) offers an ideal opportunity to investigate these dynamics, with extensive individual‐level data on fitness, behaviour, and genetic variation in a wild population experiencing natural selection. Banded mongooses are small (~1.5 kg) mammals that live in groups consisting of 10–40 adult individuals (Cant et al. 2016). In contrast to most cooperative breeders, reproductive skew is relatively low, with adults of both sexes regularly reproducing. Breeding is synchronised within each social group, with multiple females giving birth together, usually on the same night (Hodge et al. 2011). The resulting pups are raised communally by the group; unusually, there is no evidence that mothers can recognise their own pups or vice versa (Marshall et al. 2021). In our study population, individuals show limited dispersal and frequently breed within their natal group, resulting in close relatives often being among the pool of potential mates (Nichols et al. 2014). This, combined with the difficulty of recognising relatives within groups, results in high variance in inbreeding. Wells et al. (2018) used a 9‐generation deep pedigree to reveal that mild inbreeding is widespread, with many individuals (66.4%) having non‐zero inbreeding coefficients (mean pedigree inbreeding coefficient = 0.058), and an unusually high proportion of individuals (7.1%) being closely inbred (inbreeding coefficient > 0.25), resulting from full sibling or parent‐offspring matings. This inbreeding has fitness consequences for reproductive success in males (Wells et al. 2018) and homozygosity increases susceptibility to parasite infections (Mitchell et al. 2017). Nevertheless, the negative effects of inbreeding are to some extent buffered by social care (Wells et al. 2020), while evidence has also been found for inbreeding avoidance, mediated by extra‐group mating (Nichols et al. 2015) and non‐random breeding within social groups (Khera et al. 2021; Sanderson et al. 2015). This targeted mate choice may facilitate the maintenance of relatively high MHC diversity despite frequent inbreeding (Schubert et al. 2024). The availability of detailed longitudinal life‐history data for our study population, together with high variance in inbreeding, makes the banded mongoose an ideal study system for investigating effects of MHC variation on fitness components in a wild mammal population.

Here, we genotyped a total of 465 individuals for MHC‐I exons 2 and 3 and MHC‐II DRB exon 2 to capture the antigen‐binding sites of both MHC molecules. We first investigated associations between neutral genetic diversity and MHC diversity, hypothesising that they would be at least weakly positively correlated. We then used the MHC sequences to estimate three different measures of MHC functional diversity, which were combined with long‐term life history data to investigate the influence of MHC variation on three fitness measures: (i) pup survival, (ii) adult survival, and (iii) lifetime reproductive success (LRS). We hypothesised that individuals with higher MHC diversity should have increased fitness, but that the effects of the MHC could be non‐linear and sex‐specific.

## 2 Materials and Methods

### 2.1 Study Site and Field Data Collection

Data used in this study were collected on a wild population of banded mongooses inhabiting Queen Elizabeth National Park in Uganda (0°12′ S, 27°54′ E), which has been subjected to regular and systematic behavioural, life‐history and genetic data collection for over 25 years. The study area consists of approximately 10 km^2^ savannah including the Mweya peninsula and the surrounding area and the population is made up of 10–12 social groups at any one time, corresponding to approximately 250 individuals. All individuals are habituated to human observation at < 10 m (usually < 5 m), allowing us to collect detailed behavioural and life history data from each social group, which are visited every 1–4 days. To enable reliable location of our study animals, one or two adult individuals per group were fitted with a 27 g radio collar (< 2% of an individual's body mass; Sirtrack Ltd., New Zealand) with a 20 cm whip antenna (Biotrack Ltd., UK). Individual mongooses can be visually identified in the field through: (1) dye patterns in the fur created using commercial hair dye (L'Oreal, UK) for individuals less than 6 months of age, and (2) shaved fur patterns or (3) colour‐coded collars made from plastic for adults that will not grow any further. Collars and shave patterns were maintained during trapping events regularly taking place every 3–6 months as described by Cant (2000), Hodge (2007) and Jordan et al. (2010). Individuals were given either an individual tattoo or a subcutaneous pit tag under anaesthetic (TAG‐P‐122IJ; Wyre Micro Design Ltd., UK) during their first capture to allow permanent identification. Moreover, a 2 mm tail tip tissue sample was taken for genetic analysis. Individual age was calculated based on the time between the mothers' parturition date and, since deaths were not usually directly observed, the date of the disappearance of each individual from their group. Death could be distinguished from dispersal, since banded mongooses do not disperse individually, but in small groups (Cant et al. 2002) following a period of aggression from the rest of the group (Thompson et al. 2016). Lifespan was calculated as the timespan in days between the birth and death date. Data on daily rainfall in mm was obtained from the Mweya meteorological station, which is situated in the centre of the study site. Gaps in the meteorological data, which made up less than 5% of the relevant timespan, were imputed using Kalman Smoothing (Hyndman and Khandakar 2008).

### 2.2 Ethical Statement

Research was conducted under the approval of the Uganda National Council for Science and Technology, the Uganda Wildlife Authority, and the Ethical Review Committee of the University of Exeter. All research procedures adhered to the ASAB Guidelines for the Treatment of Animals in Behavioural Research and Teaching (ASAB Ethical Committee/ABS Animal Care Committee 2023).

### 2.3 Genetic Analyses

#### 2.3.1 DNA Extraction and Microsatellite Genotyping

We extracted DNA from 465 individuals using the Qiagen DNeasy blood and tissue kit according to the manufacturer's protocol. These individuals were genotyped at 35–43 microsatellite loci as described in detail by Sanderson et al. (2015).

#### 2.3.2 Heterozygosity Estimation and Parentage Assignment

Microsatellite genotypes were used to estimate individual standardised multilocus heterozygosity (sMLH) using the R package InbreedR (Stoffel et al. 2016). Outliers with sMLH values of zero or samples for which microsatellite amplification was successful for too few loci were removed. We used the R package *masterbayes* (Hadfield et al. 2006) to assign parentage, following the protocol described in (Wells et al. 2018). Briefly, parentage assignments required that candidate parents were alive and of reproductive age (> 6 months) during the relevant reproductive window, and that offspring could not be assigned as their own parents. We incorporated phenotypic data—age, recent birth records (females), and presence in the natal pack (males)—to improve assignment confidence. Assignments with posterior probability ≥ 0.8 were accepted (mean = 0.99). We used *colony* (Jones and Wang 2010) both to confirm the *masterbayes* assignments and to assign sibships among individuals with one or both unsampled parents.

#### 2.3.3 MHC Genotyping

MHC genotyping and data processing was carried out according to the protocol described in Schubert et al. (2024). In short, we amplified the MHC‐I exon 2 using primers (F: CCACTCCCTGAGGTATTTCTACACC, R: CTCACCGGCCTCGCTCTG) based on the sequences published by Yuhki and O'Brien (1990), and the MHC‐I exon 3 (F: GGTCACACAGCATCCAGAGA, R: GCTGCAGCGTCTCCTTCC) and MHC‐II DRB exon 2 (F: CGAGTGCCATTTCACCAACG, R: GCTGCACCGTGAAGCTCT) based on sequences of closely related carnivore species. All samples were amplified with a technical replicate and each plate contained a negative control. After initial amplification, samples were purified and normalised using the NGS Normalisation 96‐well kit (Norgen Biotek Corp., Canada). Libraries were then prepared using the llumina TruSeq DNA Nano Low Throughput Library Prep Kit (Illumina Inc., USA) and the TruSeq DNA Single Indexes Set A and B (Illumina Inc., USA). After checking library quality using the Agilent Bioanalyzer 2100 (Agilent Technologies, USA) together with the High Sensitivity DNA Kit (Agilent Technologies, USA), we removed primer dimers by running the libraries for 3 h at 65 V on a 1.5% agarose gel, cutting out bands corresponding to the correct amplicon sizes, and finally using either the innuPREP Gel Extraction Kit (Analytik Jena, Germany) or the QIAquick Gel Extraction Kit (Qiagen) according to the manufacturer's protocol. Libraries were then pooled and sequenced on an Illumina MiSeq (Illumina Inc., USA) with a MiSeq Reagent Kit v2 (500 cycles) according to the manufacturer's protocol at the Max Planck Institute for Evolutionary Biology, Plön, Germany. We estimated the reproducibility of the genotypes based on the formula [(Number of shared alleles × 2)/sum of alleles in replicates] to investigate consistency between the replicates. Reproducibility was 89.4% for the MHC‐I exon 2, 96.6% for the MHC‐I exon 3, and 97.7% for the MHC‐II DRB exon 2. In total, genotypes were established successfully for 321 individuals for the MHC‐I exon 2, 285 individuals for the MHC‐I exon 3, and 385 individuals for the MHC‐II DRB exon 2. Mismatched alleles were kept as putative alleles if they fulfilled the criteria for allele validation. Assigning alleles to loci was not possible, which is why heterozygosity of the MHC could not be estimated. Detailed methods for the characterisation of the MHC in banded mongooses are described in Schubert et al. (2024).

#### 2.3.4 Measures of MHC Diversity

We estimated MHC diversity for each exon separately using three measures: (1) the mean amino acid p‐distance, (2) the number of functional alleles, and (3) supertype number. Mean amino acid p‐distance is defined as the proportion of amino acid sites at which the compared sequences differ. It is calculated by dividing the number of amino acid differences by the total number of amino acid sites compared and represents the functional distance of a genotype. We calculated the mean amino acid p‐distance in R version 4.4.0 using the function p.dist of the package *phangorn*, version 2.12.1 (Schliep 2010). Since we amplified multiple loci and were not able to assign alleles to loci, we cannot include MHC heterozygosity as a measure of MHC diversity. Instead, we used the total number of functional alleles—those lacking stop codons or frameshift mutations—per individual, as well as the number of supertypes, as measures of functional diversity. We determined the number of functional alleles by translating the filtered nucleotide sequences to identify alleles with identical amino acid sequences or stop codons. These analyses were conducted in MEGA X Version 10.0.5 (Kumar et al. 2018). Functional alleles were then assigned to functionally similar supertypes based on hierarchical clustering of the amino acid characterisation matrix (Sandberg et al. 1998) estimated based on the positively selected sites (PSS) and k‐means, as conducted by (Winternitz et al. 2015). PSS were identified using the rates of non‐synonymous (dN) and synonymous (dS) mutations for each site based on two complimentary methods: FUBAR and MEME. FUBAR (Fast, Unconstrained Bayesian AppRoximation) estimates selection rates based on a Bayesian approach (Murrell et al. 2013). It detects signatures of pervasive selection by assuming constant pressure. As individual sites can experience different levels of positive and negative selection (episodic selection), methods that only detect pervasive selection will miss episodic effects. In contrast, MEME (Mixed Effects Model of Evolution) uses a mixed‐effects maximum likelihood approach to determine individual sites with signs of pervasive and episodic selection (Murrell et al. 2012). Hence, sites were classified as PSS if they were detected by either FUBAR (pervasive selection) or MEME (pervasive and episodic selection). For analysis with FUBAR, the posterior probability was set to > 0.9 as our significance threshold, with values above this threshold indicating natural selection. For analyses with MEME, the significance threshold was set to < 0.05. Both FUBAR and MEME selection inference were carried out on the Datamonkey server (Weaver et al. 2018, https://www.datamonkey.org/, last accessed: January 18, 2024).

### 2.4 Statistical Analyses

#### 2.4.1 Assessing Collinearity Among Genetic Variables

Substantial collinearity among variables can cause problems in model interpretation (including convergence issues, changes in the direction of effects, and inflated standard errors of coefficient estimates) because the effects of collinear predictors cannot be estimated independently (reviewed in Harrison et al. 2018). Thus, we performed correlation analyses to investigate whether MHC diversity measured as mean amino acid p‐distance, number of functional alleles per individual, or supertype number are strongly correlated (correlation coefficient *r* ≥ 0.7) with each other as well as with neutral genetic diversity (sMLH). To estimate the former, we calculated Pearson's correlation coefficients in R version 4.4.0 (R Core Team 2024) for correlations between MHC diversity measures. All correlations between MHC measures were highly significant, but the strength of the correlation differed for the exons. Between the MHC diversity measures, there was a strong correlation (*r* > 0.7; Table S4) between MHC‐I exon 3 mean amino acid p‐distance and number of functional alleles (*r* = 0.796, *p* < 0.001), which can be explained by the low allelic diversity of this exon. The correlation between MHC‐I exon 3 mean amino acid p‐distance and supertype number (*r* = 0.620, *p* < 0.001) as well as allele and supertype number (*r* = 0.695, *p* < 0.001) for MHC‐I exon 3 were comparably high, due to the same cause. However, correlation coefficients for the other MHC exons were often substantially above 0.2 (range −0.268 to 0.573) so we therefore decided to run separate models for each diversity measure.

To test for a relationship between MHC diversity and neutral genetic diversity (sMLH), we ran linear mixed models in R with the package *lme4* version 1.1‐35.3 (Bates et al. 2015), fitting each MHC diversity measure separately as the dependent variable and sMLH as the independent variable. We also included social group as a random slope to account for potential differences in sMLH between the social groups the individuals were born into. In effect, banded mongoose usually stay within their natal pack and rarely disperse, resulting in pack relatedness values increasing and thus individual sMLH decreasing over time (Cant et al. 2013). Hence the slope might vary depending on the age and structure of the pack an individual is born in. None of the three MHC diversity measures were strongly correlated with sMLH (*r* < 0.1; Tables S1–S3; Figures S1 and S2), so we included MHC diversity measures together with sMLH as predictor variables in the same models.

#### 2.4.2 Assessing the Impact of MHC on Fitness

We built separate models for each of the three fitness measures—pup survival, adult survival, and lifetime reproductive success—including all biologically relevant predictors and adjusting model structure as needed based on diagnostic checks. Predictors included sex, standardised multilocus heterozygosity (sMLH), rainfall, adult lifespan, social group (birth pack), litter, and birth year. The dataset included 391 unique individuals, with a male‐biased sex ratio (56% male) and representation from 6 social groups across 24 years (1994–2018). On average, 16 individuals were born per year (range: 1–52). Individuals had on average 2.8 pups over their lifetime (range: 0–77), and the average lifespan was 893 days (range: 0–5885). Monthly rainfall in the 30 days prior to birth ranged from 0 to 222 mm (mean = 70 mm), and sMLH ranged from 0.42 to 1.49 (mean = 1.00), indicating substantial environmental and genetic variation. We included sMLH to account for genome‐wide diversity, and other predictors are described in the following sections for each fitness measure.

Due to DNA quality, a subset of genotypes was missing for each MHC exon. To maximise statistical power, we analysed each MHC exon separately rather than limiting analyses to the subset of 205 individuals genotyped at all exons. As a result, we fitted nine generalised linear mixed models (GLMMs) per fitness measure. For each of the three MHC loci (MHC‐I exon 2, MHC‐I exon 3, and MHC‐II DRB exon 2), we included an MHC diversity measure—mean amino acid p‐distance, number of functional alleles, or supertype number—as the main predictor, along with its interaction with sex. We excluded 35 individuals with implausible or missing data: duplicate IDs, sMLH values of 0, unsexed, and packs smaller than three individuals. For all models, numeric predictors were scaled and centred to facilitate model convergence, allow for direct comparison of effect sizes, and reduce multicollinearity in interaction terms. All analyses were conducted in R version 4.5.0. Detailed model structures for all 27 models are provided in Table S5.

##### 2.4.2.1 Pup Survival

We included pup survival to nutritional independence (90 days) as a fitness measure because this is the most vulnerable life stage in banded mongooses, with over 50% mortality (Gilchrist 2004; Hodge 2005; Rood 1975). Survival during this period is strongly influenced by energetic condition and predation risk (Gilchrist 2004; Hodge 2005), both of which may be shaped by infection status and early immune function (Gilchrist 2004; Hodge 2005; Leclaire and Faulkner 2014; Murray et al. 1997; Shanebeck et al. 2022; Temple 1987). Furthermore, while the adaptive immune system of juvenile mammals is immature, it is nevertheless functional, gradually developing over the first few weeks of life and reaching maturity between 6 and 12 months of age (Pereira et al. 2023). Thus, even early‐life MHC effects could potentially impact survival outcomes.

We ran models analysing the association between MHC diversity and pup survival using the glmer function of the *lme4* package version 1.1‐35.3 (Bates et al. 2015). Survival to nutritional independence was modelled as the dependent variable with a binomial response (with 1 = survived, and 0 = died). As independent variables, we used one of the measures of MHC diversity in interaction with sex and sMLH as a previous study on banded mongooses found higher pup survival for offspring originating from extra‐group matings, which are more heterozygous (Nichols et al. 2015). We also included the amount of rainfall within 30 days prior to birth, as this is linked to early life survival in banded mongooses (Sanderson et al. 2015; Wells et al. 2020). To account for non‐independence among pups, the identity of the pup's litter nested within social group was included as a random effect (Wells et al. 2020). Year of birth was also included as a random effect to account for temporal non‐independence. For individual MHC allele number, we included a quadratic term to test for nonlinear effects on pup survival; this was ultimately removed as it did not significantly improve the explained variation. Model assumptions of overdispersion, singularity, collinearity, predictions matching observed data, convergence, residual normality, dispersion, and outliers were tested using the R package *performance* version 0.13.0 (Lüdecke et al. 2021) and *DHARMa* (Hartig 2024). We compared full models with corresponding null models containing only random effects and intercept to assess the contribution of the fixed effects using Bayes Factors calculated with the *performance* package in R.

##### 2.4.2.2 Adult Survival

We ran Cox proportional hazard models to analyse the potential link between MHC diversity and adult survival using the coxph function of the *survival* package version 3.6‐4 (Therneau 2024). In our models, data was right censored for individuals that either survived to the end of the study or emigrated. To account for non‐independence among individuals within social groups, we included pack as a frailty term, representing a shared random effect. Year of birth was also considered to capture temporal variation in survival, but Cox proportional hazards models permit only one frailty term. Model comparisons using Bayes Factors consistently favoured pack over year of birth, so we retained pack in all final models. The total lifespan in days was included as the dependent variable and one of the measures of MHC diversity in interaction with sex and sMLH was included as independent variables. For individual MHC allele number, we included a quadratic term to test for nonlinear effects on adult survival but finally removed it from the model as it did not significantly improve the fit. We confirmed the proportional hazards assumption using the cox.zph function from the *survival* package in R, which assesses the independence of scaled Schoenfeld residuals from time. We compared all full models to corresponding null models containing only the random effects and intercept to assess the contribution of the fixed effects using Bayes Factors calculated with the *performance* package in R.

##### 2.4.2.3 Lifetime Reproductive Success

We fit a zero‐inflated negative binomial generalised linear mixed model with Gamma priors for the random effects using the glmmTMB function [family = nbinom1, ziformula = ~1, priors = gamma(1, 2.5)] from the package *glmmTMB* version 1.1.9 (Brooks et al. 2017). We used the total number of offspring assigned in the pedigree to each individual as the dependent variable. Social group was included as a random effect to account for non‐independence among adults. We also tried to include birth year to capture temporal non‐independence among individuals, but this led to model nonconvergence and was ultimately dropped from all models. One of the MHC diversity measures in interaction with sex was included as an independent variable. Furthermore, sMLH, mean monthly rainfall per lifetime and lifespan itself were also included as independent variables. For individual MHC allele number, we included a quadratic term to test for nonlinear effects on total number of pups produced. Model assumptions were verified as explained in Section 2.4.2.1. We compared the full models with the corresponding null models containing only the random effect and intercept to assess the contribution of the fixed effects using Bayes Factors calculated with the *performance* package in R.

#### 2.4.3 Standardisation of Effect Sizes and *R* ^2^ Calculations

Effect sizes were exported from the respective models and standardised using the standardize_parameters function from the *effectsize* package version 0.8.9 (Ben‐Shachar et al. 2020) using the “refit” function. Model quality was evaluated using the R *performance* package. Nakagawa's *R* ^2^ was used for pup survival mixed models to infer the conditional variance explained by fixed and random effects, and the marginal variance explained by only the fixed effects. Nagelkerke's pseudo‐*R* ^2^, which compares the full model to a baseline model without predictors based on log‐likelihood, was used to infer the proportion of variation in adult survival and lifetime reproductive success explained by the model, scaled to a maximum of 1.

#### 2.4.4 Correcting for Multiple Hypotheses Testing

We corrected for multiple testing for each dependent variable investigated (pup survival, adult survival, LRS) using the false discovery rate (FDR) (Benjamini and Hochberg 1995). We used the p.adjust function from the *stats* package (R Core Team 2024) with “fdr” as the method of choice correcting for nine hypotheses tested for each fitness measurement (three MHC genes tested with three MHC diversity measures each). Corrections were applied within each predictor variable across the nine models to balance false discovery control with sensitivity to true effects.

## 3 Results

### 3.1 Genotyping Results

Genotyping was successful for 321 individuals for the MHC‐I exon 2, 282 individuals for the MHC‐I exon 3, and 385 individuals for the MHC‐II DRB exon 2. After removing a small number of outliers, the number of sequences per individual ranged from 2 to 11 (median = 6, *n* = 300) for the MHC‐I exon 2, between 1 and 5 (median = 1, *n* = 264) for the MHC‐I exon 3, and between 1 and 6 (median = 2, *N* = 361) for the MHC‐II DRB exon 2. Functional differences, quantified as mean amino acid p‐distances, ranged from 0.1447 to 0.333 (median = 0.257) for the MHC‐I exon 2, from 0 (only one allele per individual) to 0.182 (median = 0) for the MHC‐I exon 3, and from 0 (only one allele per individual) to 0.349 (median = 0.273) for the MHC‐II DRB exon 2. Supertype numbers ranged from 1 to 3 (median = 2) for the MHC‐I exon 2, from 1 to 2 (median = 1) for the MHC‐I exon 3, and from 1 to 2 (median = 2) for the MHC‐II DRB exon 2.

### 3.2 Associations Between MHC and sMLH

We found no evidence that any of our MHC diversity measures were significantly positively correlated with genome‐wide diversity (sMLH) based on 35–43 microsatellite markers (Tables S1–S3). This lack of association suggests that MHC diversity in banded mongooses is not primarily driven by genetic drift, but more likely maintained by balancing selection.

### 3.3 Pup Survival

**FIGURE 1:**
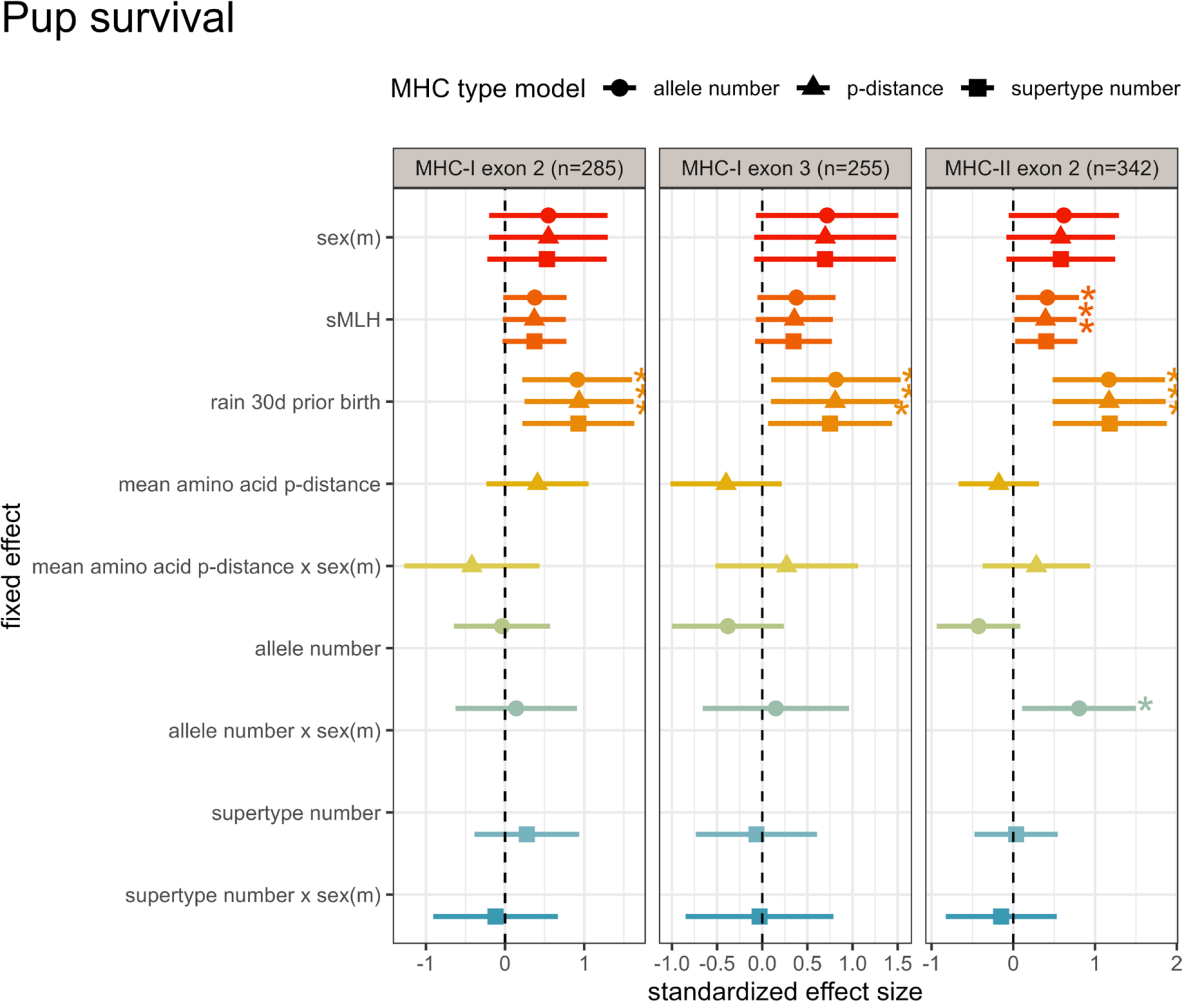
Standardised effect sizes for models of pup survival. This plot shows the standardised effect sizes of the models investigating relationships between MHC diversity and pup survival to nutritional independence (90 days). The effect sizes are shown separately for three different diversity measures (allele number, amino acid p‐distance and supertype number) for the three different genes (MHC‐I exon 2, 3 and MHC‐II DRB exon 2). Asterisks indicate significant variables before FDR correction for multiple testing.

Only one interaction showed a clear sex‐specific pattern: the effect of MHC‐II DRB exon 2 allele number on pup survival (Figure 1; Table S6). In females, each additional allele was associated with a 35% reduction in the odds of reaching independence (OR = 0.654), whereas in males, the odds of survival to independence increased by 46% per allele (OR = 1.461). Although neither slope was individually significant (Table S9), the interaction between sexes was (*p* = 0.023, Figure S3). This interaction did not remain statistically significant after FDR correction (*p*_corrected_ = 0.211), but the magnitude of the effect suggests potential biological relevance.

Rainfall in the month prior to birth consistently showed strong effects across all models, with each ~50 mm (1SD) increase associated with a 2‐ to 3‐fold increase in the odds of pup survival (OR range: 2.12–3.26, Table S6). These effects remained statistically significant after FDR correction, suggesting that wetter pre‐birth conditions enhance early‐life fitness outcomes.

Neutral genetic diversity (sMLH) in MHC‐II exon 2 models, those with the largest sample sizes, was associated with a 48%–52% increase in the odds of pup survival (OR = 1.48–1.52, Table S6), suggesting that more outbred pups are more likely to reach nutritional independence. Although these effects did not remain significant after FDR correction, 95% confidence intervals were consistently positive and right‐skewed across all nine models, suggesting weak but consistent effects.

Random effects of pack and birth year were consistently favoured over full models based on Bayes Factors and explained most of the variation in survival outcomes. Conditional *R* ^2^ values were high (~71%–77%, Table S10), while marginal *R* ^2^ values were low (6%–13%, Table S10), indicating that social pack and the timing of birth accounted for the majority of variance, with fixed effects such as rainfall, sMLH, and MHC‐II diversity contributing modestly.

### 3.4 Adult Survival

None of the MHC diversity measures, sex and their interaction, nor sMLH were significantly associated with adult survival (Table S7). However, social pack as a frailty term, representing shared variance at the group level, remained significant after FDR correction (Table S7). Overall, the full models explained only 7%–8% of the variation in adult survival (Nagelkerke's pseudo‐*R* ^2^, Table S10).

### 3.5 Lifetime Reproductive Success

**FIGURE 2:**
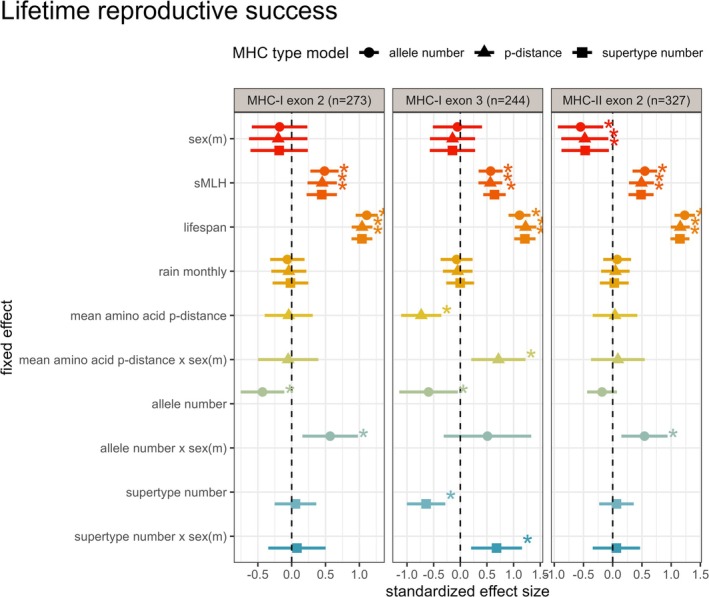
Standardised effect sizes for models investigating lifetime reproductive success. This figure shows standardised effect sizes of the models investigating links of MHC diversity with lifetime reproductive success. The effect sizes are shown separately for the different diversity measures (allele number, amino acid p‐distance and supertype number) for the three different genes (MHC‐I exon 2, 3 and MHC‐II DRB exon 2). Asterisks indicate significant initial *p*‐values before correcting for multiple hypothesis testing.

**FIGURE 3:**
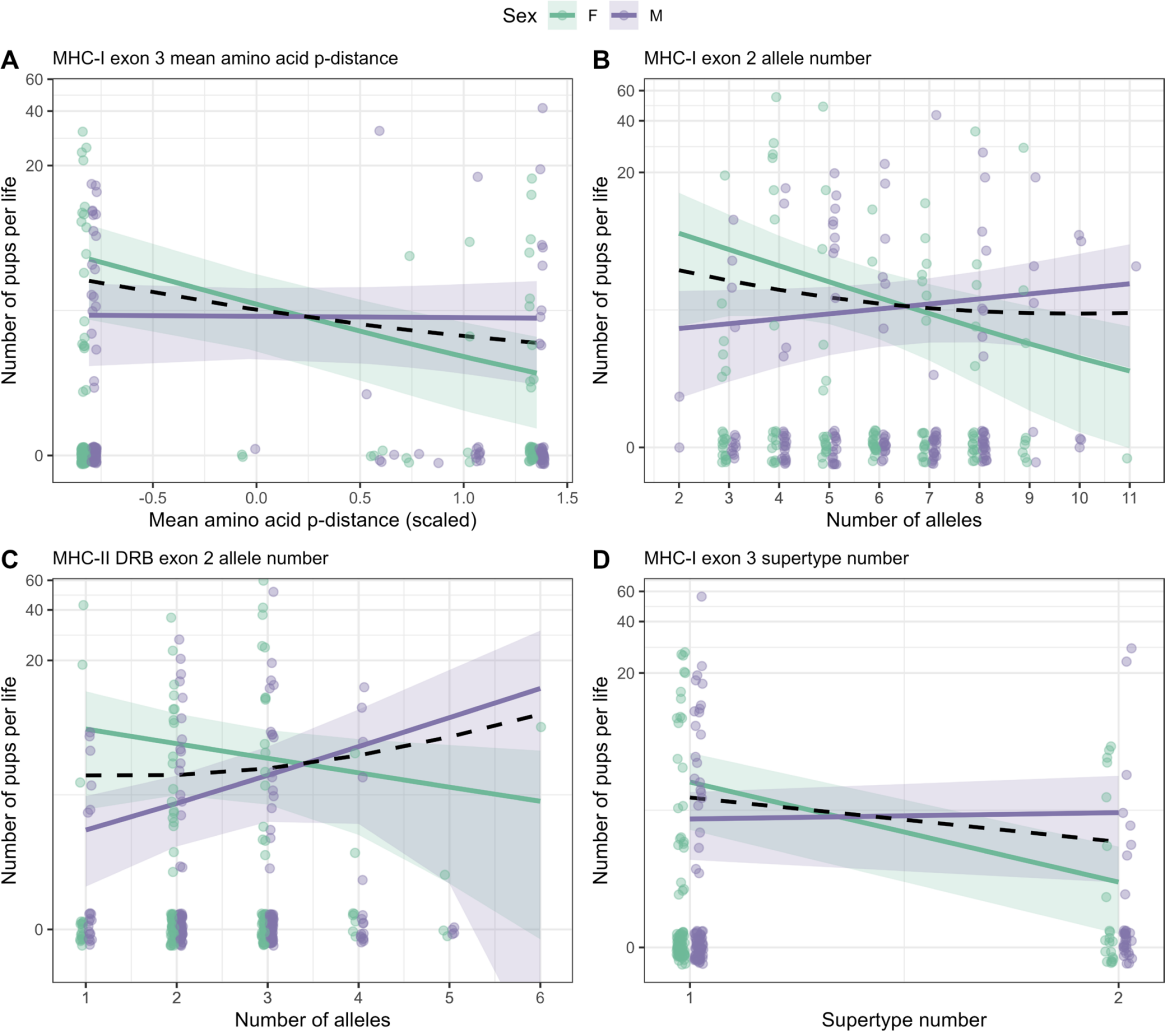
Relationship between individual MHC diversity measures and lifetime reproductive success. The investigated variables are the interaction between (A) individual MHC‐I exon 3 mean amino acid p‐distance and sex, (B) number of MHC‐I exon 2 alleles and sex, (C) number of MHC‐II DRB exon 2 alleles and sex, and (D) number of MHC‐I exon 3 supertypes and sex. Solid lines depict model predictions for each sex (males: Purple, females: Green) and the dashed black line depicts the model prediction for both sexes combined. Shaded bands show 95% confidence intervals. Expected values of the response are averaged across random effect groups and all non‐focal terms. Raw data is superimposed as coloured points. The *y*‐axis is shown on a pseudo‐log_10_ scale; 0.5 was added to all values to allow inclusion of data points with zero pups.

Males tended to have lower lifetime reproductive success compared to females (Figure 2), although only one out of the three effects remained statistically significant after correcting the *p*‐values for multiple testing (Table S8). Four of our nine interactions between MHC diversity and sex significantly predicted lifetime reproductive success after FDR correction (Figure 2; Table S8). Thus, we investigated the slopes of the interactions of MHC diversity by sex affecting lifetime reproductive success and observed contrasting directions between the sexes (Figure 3; Table S9).

Across models of lifetime reproductive success (LRS), several sex‐specific associations with MHC diversity emerged. For MHC‐I exon 3 (Figure 3A), greater mean amino acid p‐distance was associated with fewer pups over the lifetime in females (OR = 0.48, 95% CI: 0.35–0.70), while males showed no effect (OR = 0.98, 95% CI: 0.72–1.35). For MHC‐I exon 2 (Figure 3B), a higher number of MHC alleles was likewise linked to reduced female LRS (OR = 0.65, 95% CI: 0.47–0.90), but not in males (OR = 1.15, 95% CI: 0.90–1.47). For MHC‐I exon 3 (Figure 3D), greater supertype number predicted lower female reproductive output (OR = 0.53, 95% CI: 0.37–0.76), with little effect in males (OR = 1.04, 95% CI: 0.79–1.37). In contrast, MHC‐II exon 2 (Figure 3C) showed a positive association between allele number and male LRS (OR = 1.43, 95% CI: 1.08–1.90), while females showed a weaker negative trend (OR = 0.83, 95% CI: 0.65–1.07). Importantly, Poisson models revealed no significant sex differences in allele number at any of the three MHC loci (all *p* > 0.5), suggesting that observed sex‐specific fitness effects were not driven by underlying differences in diversity.

When models excluded the interaction with sex, higher MHC diversity was still associated with lower LRS—driven largely by female patterns. This was observed for MHC‐I exon 2 allele number, exon 3 amino acid p‐distance, and supertype number, highlighting the risk of reaching misleading conclusions when not explicitly modelling sex‐specific effects.

Across all models, lifespan was the strongest predictor of LRS: individuals that lived longer had more offspring. Neutral genetic diversity (sMLH) was also consistently positively associated with LRS, indicating that more heterozygous (i.e., less inbred) individuals produced more offspring than those with lower heterozygosity (Table S8; Figure 2).

Finally, full models, including MHC predictors, sex interactions, and covariates, outperformed null models based on Bayes Factors and explained a moderate to substantial portion of variation in LRS (Nagelkerke's pseudo‐*R* ^2^ = 9%–54%; Table S10).

## 4 Discussion

We found that MHC diversity had sex‐specific effects on both juvenile survival and lifetime reproductive success in banded mongooses. Males generally benefited from greater MHC diversity: lifetime reproductive success increased with allele number at MHC‐I exon 2, supertype number at MHC‐I exon 3, and allele number at MHC‐II DRB exon 2. In contrast, females showed the opposite pattern, with reproductive success declining at higher MHC diversity. A similar sex difference emerged for juvenile survival: higher MHC‐II diversity was associated with increased survival in males but decreased survival in females. Notably, there was no evidence that overall MHC diversity differed between sexes, supporting the idea of sexually antagonistic selection where the same genetic traits confer fitness benefits to one sex and costs to the other.

This sexual conflict may be particularly difficult to resolve in MHC genes. BLAST searches of our sequences against the banded mongoose genome (NCBI assembly GCA_028533875.1) indicated MHC loci are located on autosomal chromosomes (chr. 8), consistent with all other mammals studied to date (Kumánovics et al. 2003; The MHC Sequencing Consortium 1999). Because MHC alleles are inherited equally from both parents, selection acting in opposite directions in the two sexes cannot be resolved via sex‐linked inheritance. Although sex‐specific gene expression could potentially mitigate this conflict, such regulation would likely weaken the sex‐specific fitness associations we observed, making differential expression an unlikely explanation. However, this possibility warrants further investigation.

One plausible mechanism behind these sex differences lies in immune function. MHC diversity enhances the breadth of antigen presentation and T‐cell receptor repertoires, bolstering immune defence (Carrington et al. 1999; O'Connor et al. 2010; Pierini and Lenz 2018; Viard et al. 2024). However, emerging evidence suggests that the immune effects of MHC alleles differ by sex. Males and females with identical MHC genotypes can exhibit different T‐cell activation profiles (Schneider‐Hohendorf et al. 2018), and females often show stronger baseline immunity, including higher CD4+ T‐cell counts and antibody levels (Klein and Flanagan 2016). This implies that, while a diverse MHC genotype may optimally boost male immune function, it may over‐activate immunity in females, potentially leading to immunopathology or energetic trade‐offs that reduce fitness. Our results are consistent with this scenario: in the banded mongoose, MHC diversity confers greater reproductive and survival advantages in males, while imposing potential costs in females.

Support for sex‐specific costs of MHC diversity also comes from experimental studies. In mice infected with *Salmonella*, heterozygous females produced fewer offspring than homozygotes, likely due to energetic trade‐offs from heightened immune activation. This pattern was not observed in males (Ilmonen et al. 2007). In lupus‐prone mouse models, MHC heterozygosity was a prerequisite for developing autoimmune disease, especially in females (Gubbels et al. 2005). This may reflect an increased chance of self‐reactive T cells escaping deletion due to broader antigen presentation (Gubbels et al. 2005; Kretz‐Rommel and Rubin 2000), a process exacerbated in already more reactive female immune systems. Such findings may help explain why autoimmune conditions are disproportionately common in females (Kronzer et al. 2021; Whitacre 2001).

Although we lack direct infection data for this study, pathogens are known to exert strong selective pressures on banded mongooses. For example, gastrointestinal parasites are found at high prevalence in our study population (Mitchell et al. 2017), and a novel *Mycobacterium tuberculosis* complex pathogen (*M. mungi*) has caused widespread mortality across populations in Botswana, affecting nearly all tracked social groups (Alexander et al. 2018). Similar impacts have been documented in meerkats due to another TB strain, *M. suricattae* (Patterson et al. 2017). Future studies should explore links between parasitism and MHC diversity in this system.

Sex‐specific benefits of MHC diversity in banded mongooses may also be shaped by the mating system. During estrus, males compete to guard females, and dominant males usually guard older, more fecund females (Cant 2000; Nichols et al. 2010). Males with low MHC diversity may be in poorer health or condition and less capable of securing mates. This is supported by evidence controlling for age that smaller males, who also carry higher parasite loads, have reduced reproductive success (Birch et al. 2024; Mitchell et al. 2017). Similar associations between low MHC diversity, higher parasite loads, and reduced condition have been observed in other species (e.g., Lenz et al. 2009; Meyer‐Lucht and Sommer 2005), though not universally (Rauch et al. 2006).

Additionally, female mate choice may contribute to the observed sex‐specific patterns. Although females are guarded during estrus, they retain agency in mate choice and often breed with males other than their guards (Sanderson et al. 2015). Our finding of no correlation between MHC diversity and genome‐wide heterozygosity suggests that mate choice is not random with respect to MHC. If females prefer MHC‐diverse males, as documented in other mammals (Kamiya et al. 2014; Winternitz et al. 2017), this could enhance the reproductive success of such males. Such preferences are supported by findings in macaques, where more MHC‐II diverse males, but not females, have higher reproductive success (Sauermann et al. 2001).

To disentangle MHC effects from those of genome‐wide diversity, we included standardised multilocus heterozygosity (sMLH) in our models. MHC effects remained significant, indicating they are not artefacts of overall inbreeding. Interestingly, sMLH itself was a consistent predictor of lifetime reproductive success, supporting the role of inbreeding depression in shaping fitness. Prior work in this species and others has shown links between heterozygosity, body condition, and parasite load (Hoffman et al. 2014; Mitchell et al. 2017; Wells et al. 2018), underscoring the importance of genetic diversity more broadly. However, weak correlations between MHC diversity and sMLH suggest different evolutionary forces are acting on the MHC, possibly including parasite‐mediated balancing selection.

We observed a tentative sex‐specific effect of MHC‐II diversity on pup survival, which may reflect early‐life immune differences potentially driven by hormonal variation shortly after birth, as seen in rodents (Pang and Tang 1984). A caveat is that our models did not explicitly include maternal identity as a random effect. However, maternal antibody influences are likely captured by litter effects, given that communal nursing begins immediately after birth and pups suckle from multiple females (Cant et al. 2013). While maternal identity could account for in utero effects, previous work found no survival benefits of increased prenatal investment in this species (Inzani et al. 2016). Nonetheless, maternal effects may shape later‐life outcomes, and future studies linking prenatal investment, hormone levels, and MHC genotype could further clarify these early‐life pathways.

The consistency of MHC effects across our fitness proxies further strengthens our conclusions. Despite differences in sample composition, we found similar effect sizes and directions across models. The observation that MHC diversity did not predict adult survival is unlikely to be a modelling artefact, as the model diagnostics were robust and comparable to an earlier study that also obtained null results (Wells et al. 2018). Rainfall emerged as a significant predictor of pup survival and lifetime reproductive success, in line with studies in both banded mongooses and meerkats (Groenewoud and Clutton‐Brock 2021; Vitikainen et al. 2019; Wells et al. 2018). However, the bulk of variation in survival was explained by social group and year effects, highlighting the strong influence of environmental and social context.

From an evolutionary perspective, our findings support the role of sexually antagonistic selection in maintaining MHC polymorphism. If males benefit from increased MHC diversity while females incur costs, then no single MHC genotype can be optimal for both sexes. These opposing selective pressures can help preserve variation at immune loci. Although many theoretical models have explored the roles of mate choice and parasitism in shaping immune gene diversity (e.g., Bentkowski and Radwan 2019; Bentkowski and Radwan 2020; Ejsmond et al. 2014; reviewed in Winternitz and Abbate 2022), none to our knowledge have examined how much polymorphism is maintained when selection acts in opposite directions in males and females.

Sex‐specific consequences of MHC diversity are understudied but have been documented across multiple taxa. In great reed warblers, MHC‐I diversity was linked to offspring recruitment in males but had the opposite effect in females (Roved et al. 2018). In alpine chamois and macaques, MHC‐II diversity increased male lifespan and reproductive success but had no effect on females (Sauermann et al. 2001; Schaschl et al. 2012). These patterns occur in taxa with both homogametic (XX) and heterogametic (ZW) females, suggesting that incomplete sex chromosome silencing alone cannot explain why the same MHC genotype enhances male immunocompetence but overstimulates immune responses in females (Klein and Flanagan 2016). If sex hormones were the primary cause, we would expect males to benefit consistently from greater MHC diversity, given that testosterone is generally immunosuppressive and oestrogen is immunoenhancing (Foo et al. 2016; Roved et al. 2017). However, a more compelling explanation lies in resource‐based trade‐offs between mating effort and immune defence (Rolff 2002; Stoehr and Kokko 2006). This hypothesis is supported by findings of male‐biased immunity in sex‐role reversed pipefish (Roth et al. 2011) and of stronger female immune responses in insects that lack sex hormones entirely (Kelly et al. 2018).

Under this framework, the sex experiencing stronger sexual selection, often reflected in higher reproductive skew (Janicke et al. 2016), should gain more from enhanced immune function, and thus from greater MHC diversity. In banded mongooses, reproductive success is heavily skewed toward a few dominant males per pack, whereas a much larger proportion of females reproduce, suggesting that sexual selection is stronger in males (Cant 2000; Nichols et al. 2010). Conversely, in meerkats, where reproductive skew is greater among females (Clutton‐Brock et al. 2001; Ross et al. 2023), females carry higher parasite loads and may gain more from MHC diversity (Smyth and Drea 2015). Comparative studies across species with differing patterns of sex‐biased reproductive skew could provide valuable tests of these predictions.

## 5 Conclusion

Our study provides evidence for sexually antagonistic selection acting on both MHC classes in a group‐living wild mammal. By combining long‐term life‐history data with high‐throughput sequencing, we reveal contrasting, sex‐specific effects of MHC diversity on multiple fitness traits. Importantly, we analyse both MHC classes across the full peptide‐binding regions using multiple functional diversity metrics, making this one of the few studies to integrate comprehensive genetic and life‐history data in this way. Our findings underscore the importance of accounting for sex differences in studies of immunity and fitness, with broad implications for understanding how genetic variation is maintained by natural selection.

## Author Contributions

J.C.W. and N.S. were responsible for the conceptualisation of this paper. H.J.N., F.M., R.B., S.K., K.M., M.A.C. and N.S. contributed to data collection. H.J.N., J.C.W. and N.S. performed the analysis. N.S., H.J.C., J.I.H. and J.C.W. wrote the paper, and F.M., R.B., S.K., K.M. and M.A.C. revised the manuscript. All authors approved the final version of the manuscript.

## Disclosure

*Benefits generated*: In our long‐term collaboration with Ugandan researchers, we have ensured equitable benefit‐sharing by including local researchers as co‐authors, providing them with access to all data and findings, and offering training opportunities to build local research capacity. Additionally, we have shared our data and results with the wider scientific community.

## Conflicts of Interest

The authors declare no conflicts of interest.

## Supporting information


Data S1.

## Data Availability

Data and R code used in this paper can be found at the Figshare Repository: https://doi.org/10.6084/m9.figshare.28322984.
