# Supplementary material for: Sex‐Dependent Influence of Major Histocompatibility Complex Diversity on Fitness in a Social Mammal
Source: Mol Ecol. 2025 Jul 26;34(18):e70058. doi: 10.1111/mec.70058 (PMC12421486; doi:10.1111/mec.70058)
Supplement: Supplementary file 1 — Data S1. [file MEC-34-e70058-s001.pdf]

MOLECULAR ECOLOGY

Supplemental Information for:

Sex-dependent influence of major histocompatibility complex diversity on fitness in a social mammal

Nadine Schubert, Hazel J. Nichols, Francis Mwanguhya, Robert Businge, Solomon Kyambulima, Kenneth Mwesige, Joseph I. Hoffman, Michael A. Cant, Jamie C. Winternitz✉

✉ Jamie C. Winternitz
Institute for Animal Cell and Systems Biology, Evolutionary Immunogenomics, University of Hamburg, Martin-Luther-King-Platz 3, 20146 Hamburg
Email: jcwinterntz@gmail.com

Table of Contents

Table S1 Correlation analysis for mean amino acid p-distance and sMLH 2
Table S2 Correlation analysis individual MHC allele number and sMLH 2
Table S3 Correlation analysis for supertype number and sMLH 2
Table S4 Correlations between the MHC diversity measures for the data sets of each model 2
Table S5 Overview of model structure 3
Table S6 Model outputs for pup survival 5
Table S7 Model outputs for adult survival 7
Table S8 Model output for lifetime reproductive success 9
Table S9 Comparison of MHC diversity between the sexes 11
Table S10 Model fit as variance explained. 12
Figure S1 Correlation between MHC diversity measures and sMLH 13
Figure S2 Correlation between MHC diversity measures 14
Figure S3 Relationship between individual MHC-II allele number and pup survival. 15

MOLECULAR ECOLOGY

Table S1 Correlation analysis for mean amino acid p-distance and sMLH. The table displays the model output for linear mixed models of mean amino acid p-distance explained by sMLH.

MHC class and exon	*Estimate*	*CI*	*p*	*n*
MHC-I exon 2	0.00	-0.01 – 0.02	0.737	292
MHC-I exon 3	0.01	-0.04 – 0.07	0.649	260
MHC-II DRB exon 2	0.00	-0.06 – 0.06	0.929	353

Table S2 Correlation analysis individual MHC allele number and sMLH. The table displays the model output for linear mixed models of MHC allele number per individual explained by sMLH.

MHC class and exon	*Estimate*	*CI*	*p*	*n*
MHC-I exon 2	0.25	-0.93 – 1.44	0.664	292
MHC-I exon 3	0.30	-0.17 – 0.77	0.153	260
MHC-II DRB exon 2	0.05	-0.43 – 0.54	0.825	353

Table S3 Correlation analysis for supertype number and sMLH. The table displays the model output for linear mixed models of supertype number explained by sMLH.

MHC class and exon	*Estimate*	*CI*	*p*	*n*
MHC-I exon 2	0.02	-0.22 – 0.26	0.845	292
MHC-I exon 3	0.20	-0.07 – 0.47	0.147	260
MHC-II DRB exon 2	0.13	-0.13 – 0.39	0.327	353

Table S4 Correlations between the MHC diversity measures for the data sets of each model. All of the tested correlations were highly statistically significant ($P \leq 0.001$)

	Mean amino acid p-distance vs. allele number	*Mean amino acid p-distance vs. supertype number*	*Allele number vs. supertype number*
MHC-I exon 2	**-0.262*** **	**0.239*** **	**0.329*** **
MHC-I exon 3	**0.798*** **	**0.616*** **	**0.694*** **
MHC-II DRB exon 2	**0.564*** **	**0.562*** **	**0.569*** **

MOLECULAR ECOLOGY

Table S5 Overview of model structure. The table shows the model type used to investigate the effect of MHC diversity on the different fitness measures as well as the dependent and independent variables used. An interaction between the MHC diversity measure used in the model (mean amino acid p-diversity, functional allele number or supertype number of one of the genes, MHC-I exon 2 or 3 or MHC-II DRB exon 2) is fitted for each model. Furthermore, the structure of random effects is depicted. All numeric variables were centered and scaled to facilitate model convergence, allow for direct comparison of effect sizes, and reduce multicollinearity in interaction terms.

	Pup survival									*Adult survival*									*Lifetime reproductive success*								
Dependent variable	Survival to nutritional independence (90d of age)									Lifespan									Total number of offspring								
Model code	PS1	PS2	PS3	PS4	PS5	PS6	PS7	PS8	PS9	AS1	AS2	AS3	AS4	AS5	AS6	AS7	AS8	AS9	LRS1	LRS2	LRS3	LRS4	LRS5	LRS6	LRS7	LRS8	LRS9
Model type	glmer									Coxph survival model									glmmTMB								
MHC-I exon 2	x			x			x			x			x			x			x			x			x		
MHC-I exon 3		x			x			x			x			x			x			x			x			x	
MHC-II			x			x			x			x			x			x			x			x			x
mean amino acid p-distance	x	x	x							x	x	x							x	x	x						
allele number				x	x	x							x	x	x							x	x	x			
suptertype number							x	x	x							x	x	x							x	x	x
sex	x	x	x	x	x	x	x	x	x	x	x	x	x	x	x	x	x	x	x	x	x	x	x	x	x	x	x
sMLH	x	x	x	x	x	x	x	x	x	x	x	x	x	x	x	x	x	x	x	x	x	x	x	x	x	x	x

MOLECULAR ECOLOGY

Fixed effects	*MHC diversity variable* sex*	x	x	x	x	x	x	x	x	x	x	x	x	x	x	x	x	x	x	x	x	x	x	x	x	x	x	x
	rain 30d prior birth	x	x	x	x	x	x	x	x	x																		
	rain monthly																			x	x	x	x	x	x	x	x	x
	lifespan																			x	x	x	x	x	x	x	x	x
Random effects		(1 | birth pack/litter) + (1 | birth year)									(1 | birth pack)									(1| birth pack)								

MOLECULAR ECOLOGY

Table S6 Model outputs for pup survival. Summary of results from models JS1 to 9 depicting the relationships of MHC diversity and pup survival. Shown are standardized z-values, upper and lower 95%-confidence intervals, sample size and initial and corrected p-values. P-values were only corrected for relevant/significant effects using FDR correction for multiple testing. Bold values indicate significant initial p-values (p) and p-values that remained significant after correcting for multiple testing (p_{corr}).

Model code	*MHC class and exon*	*Term*	*Effect size*	*lower 0.95*	*upper 0.95*	*n*	*p*	p_{corr}
JS1	MHC-I exon 2	mean amino acid p-distance	0.410	-0.236	1.056	285	0.213	
		sex	0.548	-0.202	1.298		0.152	
		rain 30d prior birth	0.936	0.247	1.625		**0.008**	**0.015**
		sMLH	0.370	-0.028	0.768		0.069	0.108
		mean amino acid p-distance x sex	-0.420	-1.275	0.435		0.336	0.860
		groups: litter:pack, 104; birth year, 20; pack, 5						
JS2	MHC-I exon 3	mean amino acid p-distance	-0.400	-1.017	0.216	255	0.203	
		sex	0.698	-0.089	1.485		0.082	
		rain 30d prior birth	0.808	0.096	1.521		**0.026**	**0.029**
		sMLH	0.355	-0.072	0.783		0.103	0.110
		mean amino acid p-distance x sex	0.271	-0.518	1.060		0.501	0.860
		groups: litter:pack, 95; birth year, 20; pack, 6						
JS3	MHC-II exon 2	mean amino acid p-distance	-0.178	-0.670	0.314	342	0.479	
		sex	0.580	-0.085	1.245		0.087	
		rain 30d prior birth	1.172	0.480	1.865		**0.001**	**0.003**
		sMLH	0.394	0.014	0.775		**0.042**	0.108
		mean amino acid p-distance x sex	0.283	-0.375	0.941		0.399	0.860
		groups: litter:pack, 113; birth year, 20; pack, 5						
JS4	MHC-I exon 2	allele number	-0.038	-0.645	0.569	285	0.902	
		sex	0.547	-0.201	1.295		0.151	
		rain 30d prior birth	0.911	0.217	1.606		**0.010**	**0.015**
		sMLH	0.377	-0.024	0.777		0.065	0.108
		allele number*sex	0.142	-0.626	0.910		0.718	0.860
		groups: litter:pack, 104; birth year, 20; pack, 5						
JS5	MHC-I exon 3	allele number	-0.380	-1.001	0.240	255	0.229	
		sex	0.718	-0.071	1.506		0.074	
		rain 30d prior birth	0.815	0.098	1.532		**0.026**	**0.029**
		sMLH	0.379	-0.053	0.811		0.086	0.110
		allele number*sex	0.150	-0.660	0.960		0.716	0.860
		groups: litter:pack, 95; birth year, 20; pack, 6						
JS6	MHC-II exon 2	allele number	-0.425	-0.937	0.086	342	0.103	
		sex	0.618	-0.055	1.291		0.072	
		rain 30d prior birth	1.168	0.480	1.856		**0.001**	**0.003**
		sMLH	0.416	0.028	0.804		**0.036**	0.108
		allele number*sex	0.805	0.109	1.501		**0.023**	0.211
		groups: litter:pack, 113; birth year, 20; pack, 5						
JS7	MHC-I exon 2	supertype number	0.275	-0.387	0.936	285	0.416	
		sex	0.530	-0.223	1.284		0.168	
		rain 30d prior birth	0.927	0.220	1.634		**0.010**	**0.015**

MOLECULAR ECOLOGY

		sMLH	0.370	-0.033	0.774		0.072	0.108
		supertype number x sex	-0.120	-0.908	0.668		0.765	0.860
		groups: litter:pack, 104; birth year, 20; pack, 5						
		supertype number	-0.064	-0.734	0.606		0.852	
		sex	0.695	-0.089	1.480		0.082	
JS8	MHC-I exon 3	rain 30d prior birth	0.751	0.064	1.438	255	**0.032**	**0.032**
		sMLH	0.347	-0.078	0.771		0.110	0.110
		supertype number x sex	-0.029	-0.849	0.790		0.944	0.944
		groups: litter:pack, 95; birth year, 20; pack, 6						
		supertype number	0.035	-0.474	0.543		0.894	
		sex	0.582	-0.083	1.247		0.086	
JS9	MHC-II exon 2	rain 30d prior birth	1.181	0.483	1.879	342	**0.001**	**0.003**
		sMLH	0.404	0.023	0.785		**0.038**	0.108
		supertype number x sex	-0.148	-0.826	0.531		0.670	0.860
		groups: litter:pack, 113; birth year, 20; pack, 5						

MOLECULAR ECOLOGY

Table S7 Model outputs for adult survival. Summary of results from models AS1 to 9 depicting the relationships of MHC diversity and adult survival. Shown are standardized effect sizes, upper and lower 95%-confidence intervals, sample size, p-value. P-values were only corrected within terms for significant effects using FDR correction for multiple testing. Bold values indicate significant initial p-values (p) and p-values that remained significant after correcting for multiple testing (p_{corr}).

Model code	*MHC class and exon*	*Term*	*Effect size*	*lower 0.95*	*upper 0.95*	*n*	*p*	p_{corr}
AS1	MHC-I exon 2	mean amino acid p-distance	-0.122	-0.319	0.074	292	0.224	
		sex	-0.169	-0.417	0.078		0.180	
		sMLH	0.048	-0.070	0.166		0.425	
		mean amino acid p-distance*sex	0.000	-0.269	0.269		0.999	
		frailty(pack)				5 packs	**0.004**	**0.006**
AS2	MHC-I exon 3	mean amino acid p-distance	-0.171	-0.367	0.025	257	0.088	
		sex	-0.181	-0.445	0.084		0.182	
		sMLH	0.020	-0.100	0.141		0.743	
		mean amino acid p-distance*sex	0.175	-0.089	0.438		0.194	
		frailty(pack)				5 packs	**0.012**	**0.015**
AS3	MHC-II DRB exon 2	mean amino acid p-distance	-0.073	-0.240	0.095	353	0.396	
		sex	-0.150	-0.373	0.073		0.188	
		sMLH	0.052	-0.053	0.157		0.334	
		mean amino acid p-distance*sex	0.134	-0.090	0.358		0.240	
		frailty(pack)				5 packs	**0.001**	**0.003**
AS4	MHC-I exon 2	allele number	0.074	-0.114	0.261	292	0.443	
		sex	-0.147	-0.393	0.099		0.243	
		sMLH	0.048	-0.069	0.165		0.421	
		allele number*sex	-0.106	-0.353	0.141		0.401	
		frailty(pack)				5 packs	**0.003**	**0.005**
AS5	MHC-I exon 3	allele number	-0.074	-0.279	0.132	257	0.377	
		sex	-0.158	-0.421	0.105		0.194	
		sMLH	0.042	-0.080	0.164		0.730	
		allele number*sex	0.077	-0.192	0.345		0.584	
		frailty(pack)				5 packs	**0.018**	**0.018**
AS6	MHC-II DRB exon 2	allele number	-0.075	-0.237	0.087	353	0.364	
		sex	-0.150	-0.373	0.073		0.188	
		sMLH	0.057	-0.049	0.164		0.292	
		allele number*sex	0.181	-0.043	0.405		0.114	
		frailty(pack)				5 packs	**3.22E-04**	**0.001**
AS7	MHC-I exon 2	supertype number	0.003	-0.196	0.203	292	0.975	
		sex	-0.139	-0.385	0.107		0.269	
		sMLH	0.053	-0.065	0.170		0.381	
		supertype number*sex	-0.067	-0.324	0.190		0.607	
		frailty(pack)				5 packs	**0.002**	**0.005**

MOLECULAR ECOLOGY

AS8	MHC-I exon 3	supertype number	-0.099	-0.307	0.110	257	0.354	
		sex	-0.170	-0.436	0.095		0.209	
		sMLH	0.022	-0.099	0.143		0.723	
		supertype number*sex	0.093	-0.175	0.361		0.496	
		frailty(pack)				5 packs	**0.017**	**0.018**
AS9	MHC-II DRB exon 2	supertype number	-0.004	-0.177	0.168	353	0.961	
		sex	-0.147	-0.370	0.076		0.196	
		sMLH	0.049	-0.057	0.155		0.362	
		supertype number*sex	0.105	-0.119	0.330		0.357	
		frailty(pack)				5 packs	**2.84E-04**	**0.001**

MOLECULAR ECOLOGY

Table S8 Model output for lifetime reproductive success. Summary of results from models LRS1 to 9 depicting the relationships of MHC diversity and lifetime reproductive success. Shown are z-values, upper and lower 95%-confidence intervals, sample size initial and corrected and p-values. P-values were only corrected within terms for relevant/significant effects using FDR correction for multiple testing. Bold values indicate significant variables before correcting for multiple testing (p) and after multiple comparison correction (p_{corr}).

Model code	*MHC class and exon*	*Term*	*Effect size*	*lower 0.95*	*upper 0.95*	*n*	*p*	p_{corr}
LRS1	MHC-I exon 2	mean amino acid p-distance	-0.044	-0.399	0.311	273	0.809	0.843
		sex	-0.199	-0.631	0.232		0.363	0.525
		sMLH	0.451	0.234	0.668		**4.73E-05**	**5.32E-05**
		lifespan	1.040	0.887	1.193		**2.11E-40**	**3.80E-40**
		rain monthly	-0.042	-0.301	0.216		0.748	
		mean amino acid p-distance*sex	-0.052	-0.499	0.394		0.818	0.818
		groups:				5 packs		
LRS2	MHC-I exon 3	mean amino acid p-distance	-0.733	-1.110	-0.356	244	**0.000**	**0.001**
		sex	-0.146	-0.570	0.278		0.452	0.525
		sMLH	0.564	0.340	0.788		**7.99E-07**	**1.80E-06**
		lifespan	1.225	1.024	1.427		**1.08E-32**	**1.39E-32**
		rain monthly	-0.048	-0.326	0.230		0.733	
		mean amino acid p-distance*sex	0.715	0.206	1.225		**0.006**	**0.016**
		groups:				5 packs		
LRS3	MHC-II exon 2	mean amino acid p-distance	0.039	-0.343	0.421	327	0.843	0.843
		sex	-0.478	-0.880	-0.076		**0.020**	0.069
		sMLH	0.492	0.279	0.705		**6.13E-06**	**9.20E-06**
		lifespan	1.156	0.993	1.320		**1.08E-43**	**6.30E-43**
		rain monthly	0.046	-0.199	0.290		0.714	
		mean amino acid p-distance*sex	0.089	-0.371	0.548		0.705	0.818
		groups:				5 packs		
LRS4	MHC-I exon 2	allele number	-0.432	-0.754	-0.109	273	**0.009**	**0.026**
		sex	-0.179	-0.589	0.232		0.389	0.525
		sMLH	0.485	0.277	0.694		**5.19E-06**	**9.20E-06**
		lifespan	1.109	0.946	1.272		**1.83E-40**	**3.80E-40**
		rain monthly	-0.064	-0.318	0.190		0.621	
		allele number*sex	0.569	0.158	0.979		**0.007**	**0.016**
		groups:				5 packs		
LRS5	MHC-I exon 3	allele number	-0.596	-1.146	-0.046	244	**0.034**	0.076
		sex	-0.053	-0.514	0.408		0.778	0.778
		sMLH	0.569	0.343	0.795		**7.66E-07**	**1.80E-06**

MOLECULAR ECOLOGY

		lifespan	1.111	0.905	1.317		**4.02E-26**	**4.02E-26**
		rain monthly	-0.070	-0.370	0.231		0.651	
		allele number*sex	0.510	-0.311	1.331		0.223	0.401
		groups:				5 packs		
LRS6	MHC-II exon 2	allele number	-0.183	-0.439	0.073	327	0.161	0.289
		sex	-0.550	-0.939	-0.161		**0.005**	**0.045**
		sMLH	0.550	0.339	0.760		**3.06E-07**	**1.38E-06**
		lifespan	1.232	1.057	1.407		**3.07E-43**	**9.21E-43**
		rain monthly	0.076	-0.162	0.315		0.530	
		allele number*sex	0.544	0.149	0.939		**0.007**	**0.016**
		groups:				5 packs		
LRS7	MHC-I exon 2	supertype number	0.055	-0.252	0.363	273	0.724	0.843
		sex	-0.185	-0.609	0.239		0.390	0.525
		sMLH	0.445	0.220	0.669		**1.02E-04**	**1.02E-04**
		lifespan	1.039	0.884	1.193		**8.39E-40**	**1.26E-39**
		rain monthly	-0.018	-0.282	0.246		0.891	
		supertype number*sex	0.078	-0.346	0.501		0.719	0.818
		groups:				5 packs		
LRS8	MHC-I exon 3	supertype number	-0.642	-1.003	-0.281	244	**0.000**	**0.002**
		sex	-0.147	-0.573	0.278		0.467	0.525
		sMLH	0.642	0.431	0.852		**2.27E-09**	**2.04E-08**
		lifespan	1.212	1.012	1.412		**1.76E-32**	**1.98E-32**
		rain monthly	-0.002	-0.264	0.260		0.987	
		supertype number*sex	0.680	0.203	1.158		**0.005**	**0.016**
		groups:				5 packs		
LRS9	MHC-II exon 2	supertype number	0.064	-0.232	0.360	327	0.670	0.843
		sex	-0.471	-0.877	-0.066		**0.023**	0.069
		sMLH	0.485	0.269	0.700		**1.03E-05**	**1.32E-05**
		lifespan	1.150	0.987	1.313		**1.40E-43**	**6.30E-43**
		rain monthly	0.027	-0.220	0.274		0.830	
		supertype number*sex	0.063	-0.342	0.468		0.762	0.818
		groups:				5 packs		

MOLECULAR ECOLOGY

Table S9 Comparison of MHC diversity between the sexes. The table shows the slopes of MHC diversity effects separately for the sexes for the different models on juvenile survival (JS) and lifetime reproductive success (LRS). Effects with confidence intervals that do not intersect zero are in bold. Back-transformed values reflect the odds of reaching independence for per predictor unit increase for juvenile survival models and the expected count per predictor unit increase in the lifetime reproductive success models.

Model code	*MHC class & exon*	*Predictor*	*Sex*	*β*	*SE*	*Back-trans. β*	*Asymp. LCL*	*Asymp. UCL*	*n*
JS6	MHC-II DRB exon 2	allele number	F	-0.425	0.261	0.654	-0.937	0.086	143
			M	0.379	0.247	1.461	-0.105	0.864	199
LRS2	MHC-I exon 3	mean amino acid p-distance	**F**	**-0.729**	**0.191**	**0.482**	**-1.105**	**-0.354**	118
			M	-0.018	0.162	0.982	-0.335	0.299	126
LRS4	MHC-I exon 2	allele number	**F**	**-0.431**	**0.164**	**0.650**	**-0.753**	**-0.109**	123
			M	0.137	0.125	1.147	-0.109	0.382	150
LRS6	MHC-II DRB exon 2	allele number	F	-0.181	0.129	0.834	-0.435	0.072	141
			**M**	**0.358**	**0.145**	**1.430**	**0.075**	**0.642**	186
LRS8	MHC-I exon 3	supertype number	**F**	**-0.636**	**0.182**	**0.530**	**-0.992**	**-0.279**	118
			M	0.038	0.143	1.038	-0.242	0.318	126

MOLECULAR ECOLOGY

Table S10 Model fit as variance explained. Nakagawa's R^2 values for mixed models: conditional R^2 includes both fixed and random effects; marginal R^2 reflects variance explained by fixed effects. Nagelkerke's pseudo-R^2 values compare the full model to a null model (intercept-only) based on log-likelihood. They indicate the proportion of variation in the response variable explained by the fixed effects, scaled to a maximum of 1. AS models include random effects, LRS models include random effects and zero-inflation terms; pseudo-R^2 reflects fit of the fixed effects component.

Model code	*Conditional R^2*	*Marginal R^2*	*R^2 measure*
JS1	0.746	0.077	
JS2	0.722	0.073	
JS3	0.747	0.119	
JS4	0.760	0.071	
JS5	0.728	0.075	Nakagawa's R^2
JS6	0.748	0.128	
JS7	0.766	0.072	
JS8	0.713	0.064	
JS9	0.745	0.119	
AS1		0.084	
AS2		0.075	
AS3		0.080	
AS4		0.077	
AS5		0.066	Nagelkerke's pseudo-R^2
AS6		0.085	
AS7		0.077	
AS8		0.067	
AS9		0.082	
LRS1		0.541	
LRS2		0.263	
LRS3		0.201	
LRS4		0.348	
LRS5		0.388	Nagelkerke's pseudo-R^2
LRS6		0.120	
LRS7		0.539	
LRS8		0.088	
LRS9		0.217	

MOLECULAR ECOLOGY

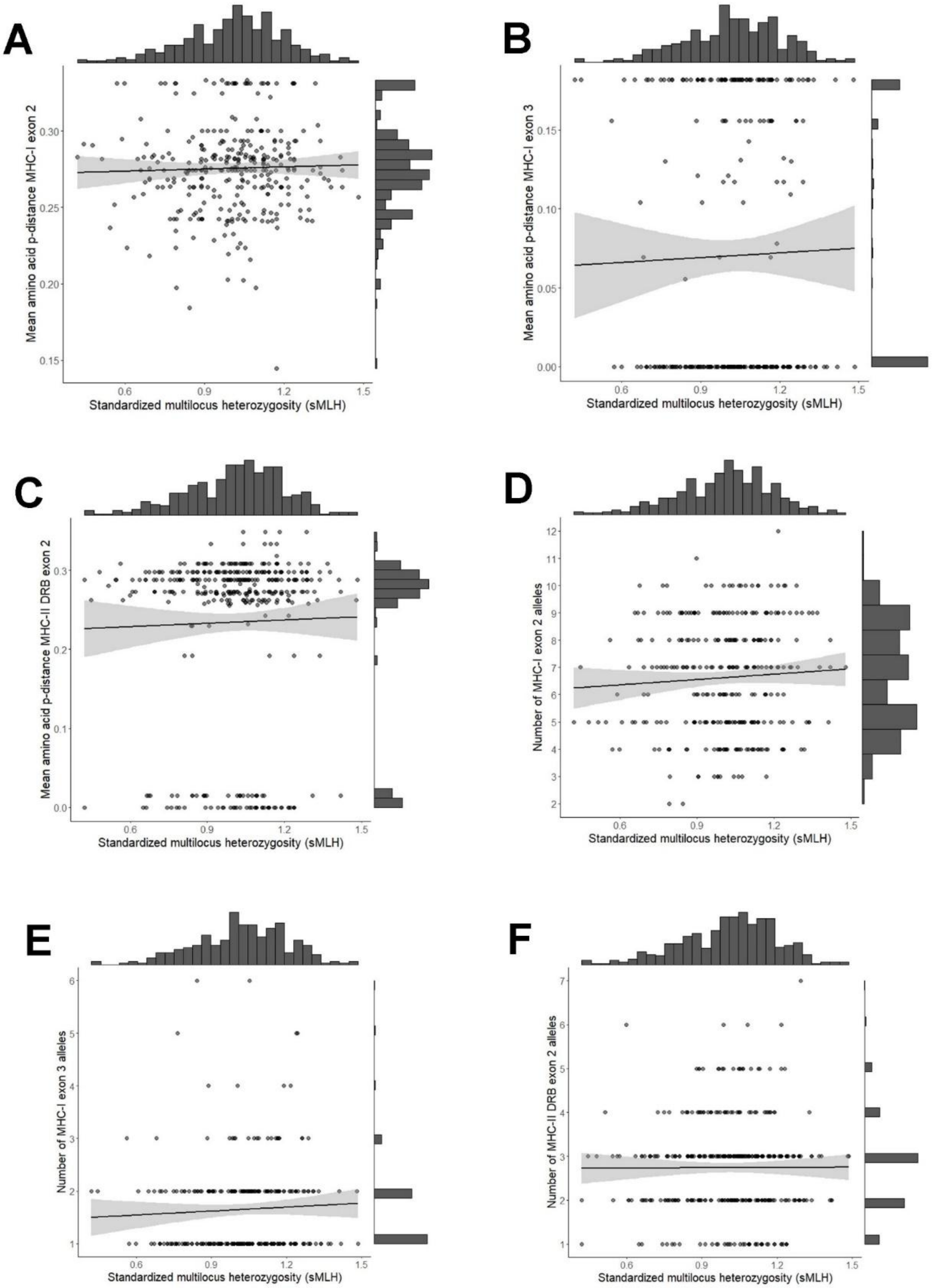

Figure S1 Correlation between MHC diversity measures and sMLH. The graphs show the raw data of mean amino acid p-distance (A-C) and individual allele number (D-F) for the three different exons plotted against the sMLH. Marginal histograms visualize the distribution of these values within the sample.

MOLECULAR ECOLOGY

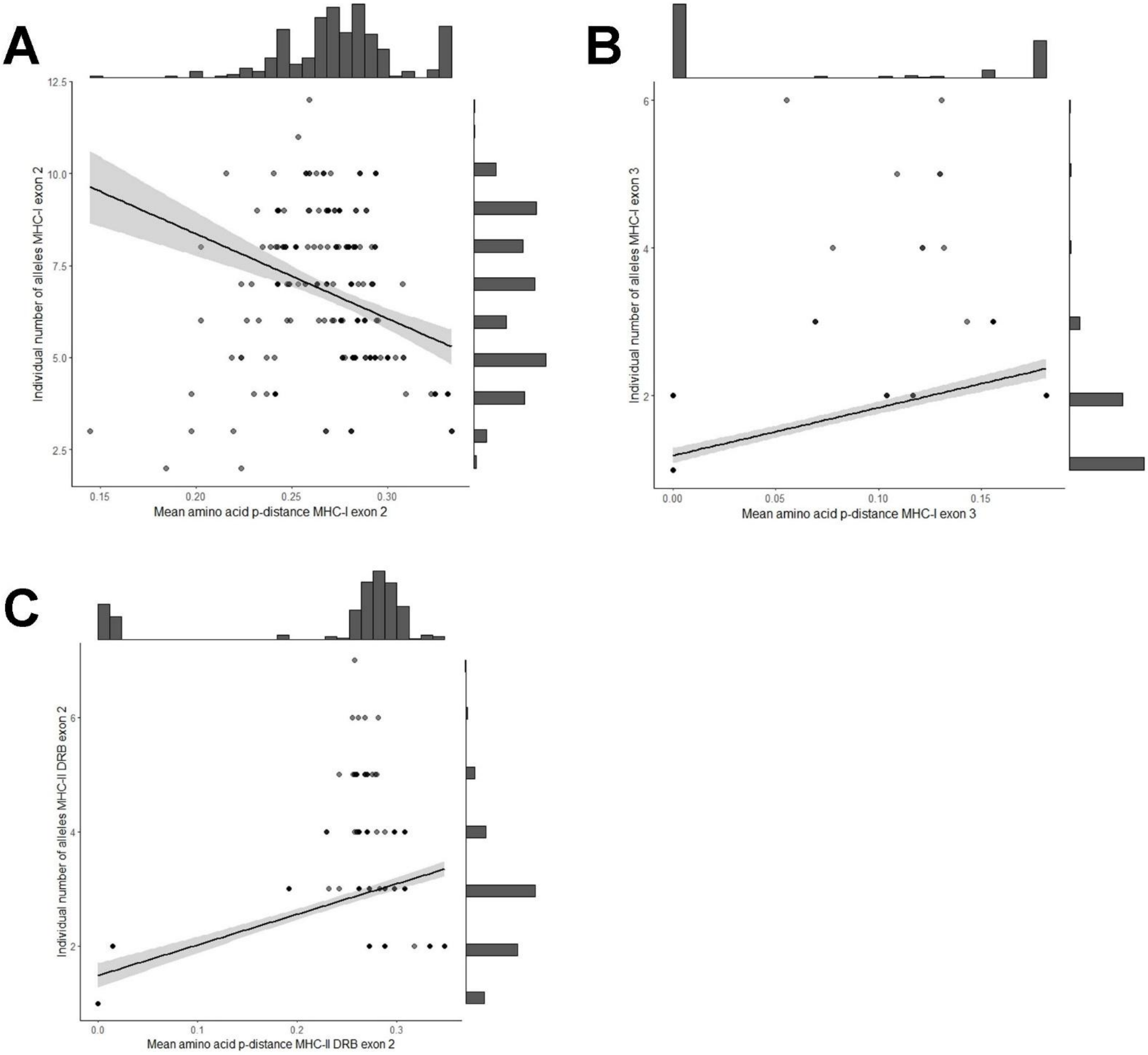

Figure S2 Correlation between MHC diversity measures. The graphs show the raw data of mean amino acid p-distance plotted against individual allele number for MHC-I exon 2 (A), MHC-I exon 3 (B), and MHC-II DRB exon 2 (C). Marginal histograms visualize the distribution of these values within the sample.

MOLECULAR ECOLOGY

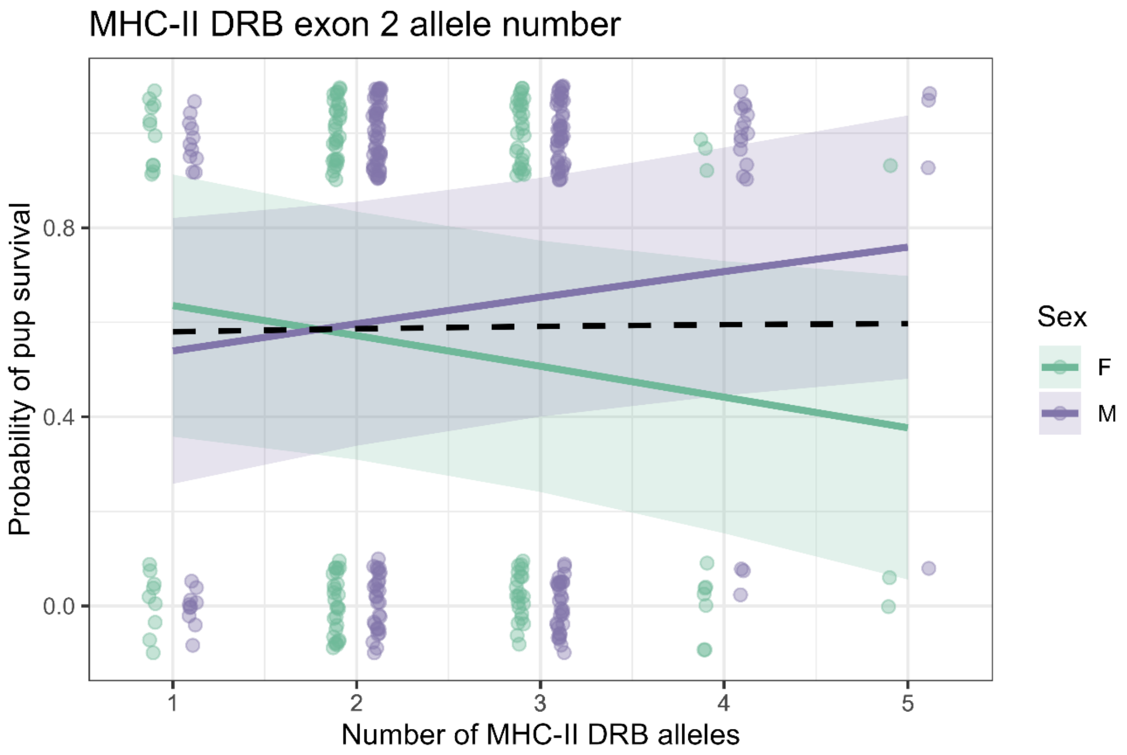

Figure S3 Relationship between individual MHC-II allele number and pup survival. Relationship between individual MHC-II DRB exon 2 allele number and survival to 90 days shown separately for the sexes. Independence is plotted as a binomial variable with 0 indicating that pups did not survive until independence and 1 indicating pup survival until independence. Regression lines are shown for females in green and males in purple, and the dashed black line represents the effect for both sexes combined. Shaded areas represent the corresponding 95% confidence intervals. Expected values of the response are averaged across all random effects groups and all non-focal terms. Raw data is superimposed as colored points (F=143, M=199, packs=5, litters=113, birth years=20).
